# Low Cost Tuberculosis Vaccine Antigens in Capsules: Expression in Chloroplasts, Bio-Encapsulation, Stability and Functional Evaluation *In Vitro*

**DOI:** 10.1371/journal.pone.0054708

**Published:** 2013-01-23

**Authors:** Priya Saikumar Lakshmi, Dheeraj Verma, Xiangdong Yang, Bethany Lloyd, Henry Daniell

**Affiliations:** Burnett School of Biomedical Sciences, College of Medicine, University of Central Florida, Orlando, Florida, United States of America; Fundació Institut d’Investigació en Ciències de la Salut Germans Trias i Pujol. Universitat Autònoma de Barcelona. CIBERES, Spain

## Abstract

Tuberculosis (TB) caused by *Mycobacterium tuberculosis* is one of the leading fatal infectious diseases. The development of TB vaccines has been recognized as a major public health priority by the World Health Organization. In this study, three candidate antigens, ESAT-6 (6kDa early secretory antigenic target) and Mtb72F (a fusion polyprotein from two TB antigens, Mtb32 and Mtb39) fused with cholera toxin B-subunit (CTB) and LipY (a cell wall protein) were expressed in tobacco and/or lettuce chloroplasts to facilitate bioencapsulation/oral delivery. Site-specific transgene integration into the chloroplast genome was confirmed by Southern blot analysis. In transplastomic leaves, CTB fusion proteins existed in soluble monomeric or multimeric forms of expected sizes and their expression levels varied depending upon the developmental stage and time of leaf harvest, with the highest-level of accumulation in mature leaves harvested at 6PM. The CTB-ESAT6 and CTB-Mtb72F expression levels reached up to 7.5% and 1.2% of total soluble protein respectively in mature tobacco leaves. Transplastomic CTB-ESAT6 lettuce plants accumulated up to 0.75% of total leaf protein. Western blot analysis of lyophilized lettuce leaves stored at room temperature for up to six months showed that the CTB-ESAT6 fusion protein was stable and preserved proper folding, disulfide bonds and assembly into pentamers for prolonged periods. Also, antigen concentration per gram of leaf tissue was increased 22 fold after lyophilization. Hemolysis assay with purified CTB-ESAT6 protein showed partial hemolysis of red blood cells and confirmed functionality of the ESAT-6 antigen. GM1-binding assay demonstrated that the CTB-ESAT6 fusion protein formed pentamers to bind with the GM1-ganglioside receptor. The expression of functional *Mycobacterium tuberculosis* antigens in transplastomic plants should facilitate development of a cost-effective and orally deliverable TB booster vaccine with potential for long-term storage at room temperature. To our knowledge, this is the first report of expression of TB vaccine antigens in chloroplasts.

## Introduction

Tuberculosis (TB) caused by *Mycobacterium tuberculosis* (MTB), is one of the leading bacterial infections that is re-emerging due to drug resistant strains worldwide. The World Health Organization (WHO) estimated the global burden of TB in 2010 to be 8.8 million cases and 650,000 cases of multi drug resistant TB (MDR-TB) [1]. TB is also the leading cause of death in HIV-infected patients as immunosuppression amplifies the risk of reactivation of TB. Bacillus Calmette Guerin (BCG), an attenuated strain of *Mycobacterium bovis* is the only available licensed vaccine against TB. Many trials that evaluated BCG on the basis of protective immunity and age of vaccination have been inconsistent and variable, ranging from 0 to 80% efficacy [2]–[5]. In adults, BCG blocks neither the development of latent TB nor revival of pulmonary TB but prevents childhood TB [6]. In mice, BCG vaccination provided protection up to 20 weeks postvaccination but the effectiveness gradually declined and was ultimately lost at 40 weeks postvaccination [7]. To confer protection, currently research groups are engaged in developing more efficient anti-TB vaccines which may have the potential to replace BCG as a primary TB vaccine or act as an effective boosting vaccine following BCG vaccination to augment protection ability [5], [6], [8]–[11]. Additional protection was not observed after BCG revaccination in randomized trials [12], [13]. Therefore, BCG itself does not execute the role of an effective booster vaccine in individuals already vaccinated with BCG or having latent TB probably for the reason that BCG induced immunity is conferred by its initial replication [6]. Thus far, only couple of replacement vaccines with higher or equal protective efficacy than BCG has emerged. Therefore, a competent prime-boost regimen strategy is to give BCG or replacement vaccine in childhood followed by an effective subunit booster vaccine at a later age. In animal models, a number of booster vaccine candidates administered at separate time gaps ranging from 15 days to 6 weeks have shown better protection than BCG alone [14]. Compared to attenuated live TB vaccines, subunit vaccines offer several advantages including safety, efficacy and are better suited for standardization [15], [16]. On the other hand, limitations include poor immunogenicity of purified antigens and restriction in the number of antigens exposed. This makes an immunostimulatory component all the more essential in an effective vaccine.

Many different elements of MTB have been proposed as subunit vaccine candidates including surface components and secreted proteins. Some of the promising antigens include ESAT-6, Ag85B, MTB72F and LipY. Any mycobacterial antigen which activates both CD4 & CD8 T- cells and imparts protective immunity is an ideal candidate for subunit vaccination against TB. Antigenic proteins actively secreted during the early phase of growth of MTB are best suited for TB subunit vaccines [17]. ESAT-6 (6kDa early secretory antigenic target) is one such promising vaccine antigen candidate that can strongly elicit a specific T-cell response [18]. ESAT-6 has been established to be present in the RD-1 region in all virulent strains of MTB but is absent in the attenuated BCG vaccine strain [19]. Hence ESAT-6 could prove to be one of the components essential to treat this complex disease. It is reported to induce production of gamma interferon (IFN-γ), a marker for protective immune response [15], [20] with protective immunity comparable to BCG [18]. The vaccines based on ESAT-6 antigen in combination with another mycobacterial antigen Ag85B have entered human clinical trials [21]. Another attractive vaccine antigen candidate is Mtb72F, a recombinant fusion polyprotein from two known TB antigens Mtb32 and Mtb39. Mice immunization with the recombinant protein Mtb72F, formulated in two different adjuvant systems - AS01B and AS02A (GlaxoSmithKline Biologicals proprietary adjuvants), resulted in the induction of strong immune response [16]. It was also reported that vaccinated mice were protected against aerosol challenge with a virulent strain of MTB [22]. In addition, vaccination with Mtb72F formulated in AS02A or AS01B protected mice against the central nervous system (CNS) challenge with *Mycobacterium tuberculosis* H37Rv [16].

LipY, a TB cell wall protein and lipase, is up-regulated during dormancy and active mycobacterium infection of macrophages. Serum from tuberculosis patients has already been shown to cause a humoral (IgG and IgM) response against LipY. Therefore, LipY may serve as a B-cell antigen. As lipases are active in M tuberculosis’s latent phase, drugs and vaccines can be targeted to latent phase cells, a current limitation to M. tuberculosis treatment, which requires an active infection [23], [24].

Mycobacterial antigens as subunit vaccines have been targeted by different delivery systems including recombinant viral vector system [25], recombinant bacterial vector system [26] and lipoglycan - protein conjugate system [27]. In this study, we developed an oral delivery system for tuberculosis antigens to be targeted to the gut associated lymphoid tissue (GALT) – an integral part of the mucosal immune system. As M. tuberculosis is a respiratory pathogen, subunit vaccination targeting the mucosa is well suited to initiate both mucosal and systemic immune response. In order to survive the extreme physiological conditions of the gut, the antigens are protected via bio-encapsulation within plant cells [28], [29].

Cholera toxin B subunit is a well-known mucosal adjuvant that has been reported as carrier molecule for mucosal immune responses [30]. It has been reported that adjuvanticity of CTB increases when coupled with foreign antigens. This could be attributed to better uptake of antigen across the mucosal linings and efficient antigen presentation to dendritic cells and macrophages. The type of the antigen fused to CTB plays an important role in the outcome of immune response [31]. ESAT-6 and Mtb72F are established immunodominant T-cell antigens. Therefore, CTB fused with TB antigens could potentiate systemic and mucosal immune response. Plant vaccines are highly efficient delivery vehicles as they are capable of transporting antigen in an encapsulated form. In animal trials, many plant oral vaccines with foreign proteins fused to CTB expressed in chloroplasts have shown to be protected against degradation by stomach enzymes and offer protective immunity against disease states [29], [32], [33].

Recombinant ESAT-6 has been expressed in transient plant production systems such as potato virus X vector based system (PVX) in tobacco plants, tobacco mosaic virus vector based system and *Agrobacterium* mediated transformation in *Arabidopsis* plants but none of them are suitable for oral delivery [34]–[36]. Expression of the TB antigens in plants through chloroplast transformation has several advantages over other production systems. Transplastomic plants lack transgene silencing in spite of accumulating >100-fold higher transcripts than nuclear transgenic plants [37], [38]. High levels of expression (up to 72% of total leaf protein) of several biopharmaceutical proteins and vaccine antigens have been attained via the chloroplast system [39]–[41]. Hyper-expression of the biopharmaceuticals in chloroplast facilitates cost-effective production without expensive purification methods and cold storage/transportation. Plant chloroplasts facilitate formation of disulfide bonds [33] and other post-translational modifications [42]. The functionality of chloroplast-derived vaccine antigens has been validated by several assays including the macrophage lysis assay [43], GM1-ganglioside binding assay [32], [44] and immune response to challenge by pathogens [29] or toxins [43]. In comparison to mammalian expression systems, there is minimal risk of animal or human pathogens contamination [43]. Additionally, chloroplast genomes facilitate transgene containment preventing escape through pollen because of maternal inheritance of the plastid genome [37], [45]. Also, total elimination of any reproductive structures is achieved when therapeutic proteins are harvested from leaves.

In this study, ESAT-6 or Mtb72F fused with CTB and LipY was expressed in tobacco as a model system for production, followed by development of CTB–ESAT6 and LipY expression in lettuce chloroplasts for oral delivery of TB vaccine antigens. The lyophilization performed on lettuce leaves provided stability, long term storage of antigens and increase in CTB–ESAT6 antigen content by 22 fold with proper folding, disulfide bonds formation and assembly into pentamers. GM1 ELISA assay confirmed binding affinity of CTB–ESAT6 to GM1 receptors. Hemolysis assay demonstrated dose dependent hemolytic activity of CTB–ESAT6 in red blood cells.

## Materials and Methods

### Construction of Chloroplast Transformation Vectors

The ESAT-6 and LipY sequence was amplified using sequence-specific restriction-site flanking primers and *Mycobacterium tuberculosis* genomic DNA as template. The PCR product was then cloned into the pCR BluntII Topo vector (Invitrogen) and sequenced to check any errors. Following *Sma*I/*Xba*I digestion, the ESAT-6 gene was ligated into the pLD Ctv 5CP chloroplast transformation vector [33] to create pLD-CTB-ESAT6. The LipY sequence was released by *Nde*I/*Xba*I digestion and ligated to pLD-Utr vector to construct pLD-LipY. The CTB sequence was amplified using sequence specific primers and pLD Ctv 5CP [33] vector as the template. Mtb72F [22] was generated by amplifying each individual fragment (Mtb32c^192–323^, *Sma*I at 5′ end and *Bam*HI at 3′ end; Mtb39, *Bam*HI at 5′ end and *Eco*RI at 3′ end; and Mtb32n^1–195^, *Eco*RI at 5′ end and *Hind*III at 3′ end) from *Mycobacterium tuberculosis* genomic DNA using sequence specific primers and sequentially linking in tandem the 14-kDa C-terminal fragment of mtb32 to the full-length fragment of mtb39, followed by the 20-kDa N-terminal portion of mtb32. Further, the sequences were confirmed to verify any errors and assembled as CTB-Mtb72F into the pBSSK+ (Stratagene, La Jolla, CA, USA) vector. The CTB-Mtb72F was then sub cloned into the tobacco chloroplast transformation vector to obtain pLD-CTB-Mtb72F.

Lettuce flanking sequence vector (pLSLF) was used to integrate the transgene into transcriptionally active spacer region between *trn*I and *trn*A genes as explained previously [33], [46]. The CTB-ESAT6 sequence was assembled and subcloned in pDVI-1 vector [40]. LipY sequence was also subcloned in pDVI-1 vector. The CTB-ESAT6 and LipY expression cassettes regulated by *psb*A promoter, 5′ and 3′ Utr was then ligated into the pLsDV vector to make lettuce chloroplast transformation vectors pLsDV-CTB-ESAT6 and pLsDV-LipY. To facilitate proper folding of protein, both fusions (CTB-ESAT6 and CTB-Mtb72F) had the GPGP hinge in between fusion proteins for reducing the steric hindrance. In this study, LipY was not fused to CTB. Oral delivery of plant cells expressing subunit vaccine candidates, without fusion to CTB has been shown to elicit immunogenic response and/or confer protection against pathogen challenge [29], [47]–[49]. Therefore, LipY was first expressed without CTB fusion but future studies will consider using CTB fusion.

### Bombardment and Selection of Transgenic Plants

Chloroplast transformation including bombardment and regeneration was carried out as described previously [40], [46]. In brief, sterile fully expanded leaves placed on MS medium with abaxial side up for tobacco (*Nicotiana tabacum* var Petite Havana and LAMD-609) and adaxial side up for lettuce (*Lactuca sativa* cv Simpson Elite*)* were bombarded with gold particles coated with plasmid DNA of pLD-CTB-ESAT6, pLD-CTB-Mtb72F, pLD-LipY, pLsDV-LipY and pLsDV-CTB-ESAT6 respectively using the biolistic device PDS1000/He (Bio-Rad). After incubation at 25°C in the dark for 2 days, the leaves were cut into small (∼5 mm^2^) pieces and placed on the regeneration medium of plants (RMOP) containing spectinomycin dihydrochloride 500 mg/l (for Petite Havana), 200 mg/l (for LAMD) and modified LR regeneration medium of lettuce prepared with spectinomycin dihydrochloride 50 mg/l (for lettuce) with bombarded side facing the medium [40]. The transgenic shoots appeared after about 4–8 weeks and were screened for transgene integration by PCR as described previously [46]. PCR positive shoots underwent an additional selection on their corresponding regeneration medium to achieve homoplasmy and were rooted in half-strength MS medium containing spectinomycin of concentrations mentioned earlier. Rooted plants were transferred to Jiffy peat pots and placed in incubator for acclimatization. After considerable growth, plants were moved to green house for maturation and seed production. In order to confirm maternal inheritance, seeds harvested from self-pollinated CTB-ESAT6 transplastomic lettuce plants were germinated on ½ MS salt supplemented with spectinomycin (100 mg/l for lettuce). Sterilization of seeds was performed with 25% bleach, followed by thorough rinsing in distilled water. Seeds from untransformed plants were also inoculated in the same plate. More than ten plates were screened with 15–20 seeds per plate. The plates were observed after 10 days.

### Confirmation of Transgene Integration into the Chloroplast Genome by Southern Blot Analysis

To confirm the transgene cassette(s) integration into the chloroplast genome, genomic DNA was extracted from leaf tissues of spectinomycin resistant and wild-type untransformed plants using Qiagen DNeasy Plant Mini Kit (Qiagen, Valencia, CA). Southern blot analysis was carried out to confirm site specific integration and to determine homoplasmy as described previously [46], [50]. Regenerated chloroplast transgenic lines were analyzed to determine whether homoplasmy was attained with all the copies of the chloroplast genome containing stably integrated transgene. In brief, 2–5 µg of genomic DNA of both tobacco (CTB-ESAT6 and CTB-Mtb72F) and lettuce (CTB-ESAT6) were digested completely with *Hind*III enzyme and run on a 0.7% (w/v) Tris-Acetate EDTA (TAE) agarose gel and transferred to a nylon membrane (N^+^-Bond, Amersham Biosciences, USA) by capillary action. Similarly, LipY tobacco genomic DNA was digested with *Sma*I and transferred to nylon membrane. The P^32^ labeled flanking sequence probe was generated and used for Southern blot analysis of transplastomic plants. Pre-hybridization and hybridization were carried out using hybridization solution (Stratagene QUICK-HYB, La Jolla, CA) according to manufacturer’s protocol. The membrane was exposed to X-ray film in a cassette at −80°C for 2 days.

### Detection of Fusion Protein Using Western Blot Analysis

Transformed and untransformed leaves (∼100 mg) were ground in liquid nitrogen with a mortar and pestle followed by extraction with a mechanical pestle in 200 µl of plant extraction buffer (200 mM Tris-HCl, pH 8.0, 100 mM NaCl, 100 mM DTT, 0.1%SDS, 400 mM sucrose, 0.05% Tween 20, 2 mM PMSF and proteinase inhibitor cocktail (Roche). Some portion of the homogenized material was aliquoted for soluble and insoluble portions of the protein extract. The leaf extracts were then centrifuged for 5 min at 10,000 rpm to separate out the supernatant containing soluble fraction and pellet the insoluble plant material. The pellet containing insoluble proteins was resuspended in buffer and sonicated in ice for 10 s pulse for one min. Bradford assay was performed to detect total protein concentration using Protein Assay Dye Reagent Concentrate (Bio-Rad). Standard curve for this assay was generated with Bovine Serum Albumin (BSA) with dilutions ranging from 0.025 mg/ml to 0.8 mg/ml. All samples were loaded in duplicate and absorbance was measured at 595 nm. Homogenate, supernatant and pellet fractions were boiled for 5 min in sample buffer (0.5M Tris-HCl, 25% glycerol, 10% SDS, 0.5% Bromophenol blue and β-mercapto ethanol) and separated by sodium dodecyl sulfate - polyacrylamide gel electrophoresis (SDS-PAGE) (Bio-Rad). The separated proteins were then transferred on to a nitrocellulose membrane in a transfer cassette (Bio-Rad) at 85V for 1 hour. After blocking with phosphate-buffered saline (PBS), 0.1% Tween 20, 3% milk powder (PTM), the membrane was incubated with anti-CTB primary antibody (1∶4000, Sigma, St. Louis, MO, USA) diluted in PTM followed by incubation with 1∶5000 horseradish peroxidase (HRP) - conjugated goat anti-rabbit secondary antibody (Southern biotech, Birmingham, AL, USA) for 1 hour 30 minutes. LipY protein was detected with rat anti-LipY (ΔPE) serum. A Super Signal West Pico HRP Substrate Kit (Pierce, Rockford, IL, USA) was used for autoradiographic detection.

### Enzyme Linked ImmunoSorbent Assay (ELISA)

To quantify the expression of the fusion protein, the enzyme linked immunosorbent assay (ELISA) was performed. Approximately 100 milligram (mg) of the leaf samples at different developmental stages (young, mature and old) or at different time points (10 AM, 12 PM and 6 PM) were collected from plants grown in the greenhouse exposed to sunlight. The extraction buffer described above was used to isolate total leaf protein for this assay. The CTB (sigma) protein standards and tested samples were diluted in the coating buffer (15 mM Na_2_CO_3_, 35 mM NaHCO_3_, 3 mM NaN_3_, pH 9.6) with the concentration from 50 to 1000 ng/mL and coated on to a 96 well ELISA plate overnight at 4°C. Blocking was performed with PTM for 1 hour. The anti-CTB primary antibody was used at 1∶4000 dilution for 1 hour. Washes were performed with 1x PBS, 0.1% tween (PBST) thrice followed by washes with distilled water. HRP conjugated goat anti-rabbit secondary antibody was used at 1∶5000 dilution for 1 hour. Washes were performed as mentioned above. Wells were then loaded with 100 µl of 3, 3, 5, 5-tetramethylbenzidine (TMB; American Qualex) substrate and incubated for 10 to 15 min at room temperature. The reaction was terminated by adding 50 µl of 2N H_2_SO_4_ per well, and the plate was read on a plate reader (Dynex Technologies) at 450 nm.

### Densitometry Analysis

To quantify the expression of fusion protein in transplastomic CTB-ESAT6 lettuce plants, densitometric analysis was performed on the immunoblots with CTB antibody. Known concentrations of purified CTB (Sigma) were used as standards (25, 50, 75 and 100 ng) to create a standard curve. Total protein concentration was measured using Bradford assay (Bio-Rad). Fusion protein was loaded in different concentrations of total protein and their Integrated Density value (IDV) was measured using Alpha imager 2000 and analyzed using Alphaease software. The percentage of fusion protein (% Total leaf protein) and amount of transgenic protein (µg/g) is calculated based on the formula published earlier [46].

### Lyophilization

Lettuce leaves expressing CTB–ESAT6 were frozen in liquid nitrogen and then lyophilized in Freezone Benchtop Freeze Dry Systems (Labconco) in vacuum (0.036 mBar) for 2 days at −50°C. Lyophilized leaves were stored at room temperature in vacuum for few days and ground to fine powder in waring blender. Ground lyophilized leaf tissue was normalized with fresh leaf tissue based on its dry weight. Immunoblots were performed with 5 mg of lyophilized tissue and 100 mg of fresh tissue since we observed 95% decrease in weight due to removal of water content. The GM1 ELISA binding assay was performed as described below to confirm stability of fusion protein in lyophilized tissue.

### GM1 - Ganglioside Receptor Binding Assay

GM -1 ganglioside (Sigma G-7641) and BSA (control) was coated at a concentration of 3 µg/ml in bicarbonate buffer (15 mM Na_2_CO_3_, 35 mM NaHCO_3_, pH 9.6) on to a 96 well plate at 4°C overnight. Washing was performed thrice with 1X PBST and water. The wells were then blocked with 200 µl of PTM for 2 hours at 37°C followed by washing thrice with 1x PBST and water. Untransformed plant leaf TSP, transformed leaf TSP (extracted in bicarbonate buffer) and CTB protein standards diluted in ELISA coating buffer were coated on to the plate in different concentrations and incubated for 2 hours at 37°C. The plate was washed again as stated above and incubated with anti - CTB primary antibody (1∶3000 dilution) for 1 hr at 37°C. Further, the plate was incubated with secondary HRP-conjugated goat anti-rabbit IgG in 1∶4000 dilution. Following washing with 1X PBST and water thrice, 100 µl of TMB substrate was added and incubated for 10 to 15 min at room temperature. The reaction was terminated by adding 50 µl of 2 N H_2_SO_4_ per well and the absorbance was read on a plate reader at 450 nm.

### Affinity Purification

Total leaf protein from CTB–ESAT6 lettuce transplastomic plants was extracted with 2X PBS (pH 8) containing 0.1% Tween 20 and proteinase inhibitors by sonication (Sonicator 3000 Misonix) for 1 min in ice. Supernatant was obtained after centrifuging for 5 min at 2000 rpm. Pre-clearing of the supernatant was performed with 100 mg of Protein A Sepharose CL-4B (GE Healthcare) beads along with protease inhibitors (Roche) overnight at 4°C. Rabbit anti-cholera toxin B subunit polyclonal antibody (Abcam, ab34992) was coated on to washed protein A beads in a ratio of 50 µg/200 µl in 1X PBS overnight at 4°C for antibody binding to beads. Washes were performed with 1X PBS followed by 0.1M sodium borate buffer (pH 9). Dimethyl pimelimidate - DMP (20mM, Sigma D8388) cross linker in sodium borate buffer was added to the bead- antibody mixture and incubated for 30 min at room temperature twice. Washes were continued with 50 mM glycine (pH 2.5) followed by 1X PBS. Cross linked antibody was added to the precleared supernatant and incubated overnight at 4°C. Elutions were performed with 100 µl glycine buffer (100 mM, pH 2.5) containing 10 µl of 1M Tris-HCl (pH 9) for neutralization at room temperature. All elution fractions were analyzed by Bradford assay (Bio-Rad) and nanodrop spectrophotometer via absorbance at 280 nm for total protein concentration in mg/ml. Silver staining was performed to detect % purity.

### Silver Staining

SDS-PAGE gel (12%) was fixed using a fixative (50% methanol, 12% acetic acid) overnight at 4°C and washed twice with 50% ethanol. The gel was pretreated with 0.02% sodium thiosulphate solution (Na_2_S_2_O_3_) for 1 min and then washed thrice in distilled water for 1 min each. Gel was stained with Silver nitrate solution (0.2% silver nitrate in formalin) for 20 min. The gel was rinsed twice in distilled water for 1 min and developer solution (2% Na_2_CO_3_, 0.0004% Na_2_S_2_O_3,_ formalin) was added. Gel was shaken to observe bands in developer solution. Developer solution was replaced after 5 min and reaction was stopped with 1% acetic acid as soon as protein bands were observed.

### Hemolysis Assay

To test the hemolytic activity of the ESAT-6 protein, hemolysis assay was performed with affinity purified CTB–ESAT6 fusion protein. Pore formation in red blood cell membranes can be measured by hemolysis assay as shown previously [51]. Affinity purified CTB – ESAT-6 was solubilized with 0.1M KCl – HCl for 1 min followed by neutralizing with 50 mM Tris - HCl (pH 8) as described earlier [52], [53]. After solubilization, CTB-ESAT6 fusion protein was mixed in 1∶2 ratio with sheep red blood cells (1×10^9^ cells) in a microcentrifuge tube and incubated at 32°C for 2 hours. Non solubilized CTB-ESAT6, distilled water and PBS incubated with sheep red blood cells in the same ratio and under same conditions were used as controls. The cells were then resuspended and centrifuged for about 7 min at 4000 rpm. Supernatants were loaded on to 96 well plate and absorbance of the supernatants was read at 405 nm.

## Results

### Tobacco and Lettuce Chloroplast Vectors Containing TB Antigens

**Figure 1:**
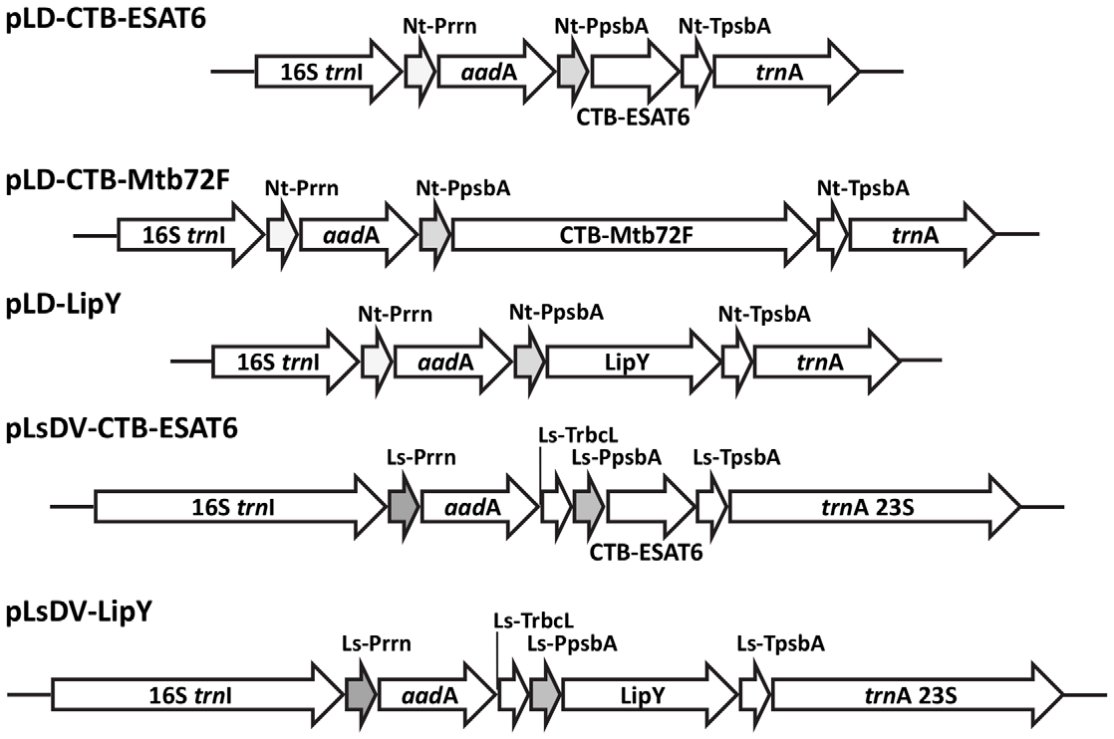
Schematic representation of chloroplast transformation vectors harboring tuberculosis candidate vaccine antigens. Ls, *Lactuca sativa*; Nt, *Nicotiana tabacum*; pLD- vector components: the homologous *Nicotiana tabacum* chloroplast genome flanking sequence comprising of 16S 3′ end sequences and complete *trn*I, *trn*A genes; pLs- vector components: 16S *trn*I, the homologous *Lactuca sativa* chloroplast genome long flanking sequence containing 16S 3′end sequences and full length *trn*I gene; *trn*A 23S, *trn*A gene and 5′end of the 23S ribosomal RNA subunit; Prrn, ribosomal RNA operon promoter with GGAGG ribosome binding site; *aad*A, aminoglycoside 3′-adenylytransferase gene; TrbcL, 3′ untranslated region (Utr) of *rbc*L gene; PpsbA, promoter and 5′ Utr of *psb*A gene; TpsbA, 3′ Utr of *psb*A gene; CTB-ESAT6, coding sequence of cholera toxin B subunit fused to ESAT6 (6 kDa early secretory antigenic target); CTB-Mtb72F, coding sequence of cholera toxin B subunit fused to a recombinant fusion polyprotein from two TB antigens Mtb32 and Mtb39; LipY, coding sequence of a TB cell wall protein with lipase activity.

Tobacco chloroplast vectors pLD-CTB-ESAT6, pLD-CTB-Mtb72F and pLD-LipY were constructed with CTB-ESAT6, CTB-Mtb72F and LipY coding sequences as subunit vaccine candidates (Figure 1). The lettuce chloroplast vectors pLsDV-CTB-ESAT6 and pLsDV-LipY harboring CTB-ESAT6 and LipY respectively were also created (Figure 1). Both CTB-ESAT6 and CTB-Mtb72F contained GPGP (Gly Pro Gly Pro) hinge at the junction of fusion proteins to assist in correct folding of each protein by reducing the steric hindrance. The tobacco vectors contained homologous flanking sequences 16S/*trn*I and *trn*A whereas lettuce vectors had longer flanking sequences containing 16S/*trn*I and *trn*A/23S (Figure 1) to facilitate recombination with the native chloroplast genome. The expression of CTB-ESAT6, CTB-Mtb72F and LipY was regulated by the endogenous *psb*A promoter and 5′ untranslated region (Utr) to achieve higher levels of expression due to the presence of multiple ribosome binding sites [40]. The *psb*A 3′ Utr (TpsbA) located at the 3′ end of the introduced gene cassette, conferred transcript stability [54]. The endogenous constitutive 16S rRNA operon promoter (Prrn) was employed to regulate expression of the *aad*A (aminoglycoside 3′ adenylyltransferase) gene with a GGAG ribosome binding site upstream of the start codon AUG to confer spectinomycin resistance. The 3′ Utr of *rbc*L gene (TrbcL) downstream of *aad*A gene provided transcript stability to *aad*A in lettuce vectors. The final chloroplast transformation vectors pLD-CTB-ESAT6, pLD-CTB-Mtb72F, pLD-LipY, pLsDV-CTB-ESAT6 and pLsDV-LipY were sequenced and used for transformation studies.

### Analysis of Transplastomic Plants for Site-specific Transgene Integration

A total of 9 (per 30 bombardments) and 26 (per 40 bombardments) independent spectinomycin resistant tobacco shoots were obtained with pLD-CTB-Mtb72F and pLD-CTB-ESAT6 vectors, respectively. Four independent spectinomycin resistant lettuce shoots were recovered from leaves bombarded with pLsDV-CTB-ESAT6 vector coated on gold particles. Comparable numbers of transplastomic tobacco and lettuce shoots were also obtained with LipY harboring vectors. Site-specific transgene integration of independent spectinomycin resistant shoots was verified by polymerase chain reaction (PCR) using 3P/3M and 5P/2M primer pairs in tobacco and 16SF/3M and 5P/2M primer pairs in lettuce (data not shown) as described previously [46]. Untransformed plants did not show any PCR product. Nuclear transformants could be distinguished because 3P or 16SF will not anneal and mutants were identified because 3M will not anneal and thus shoots that have nuclear integration or spontaneous mutation of the 16S rRNA gene were eliminated.

**Figure 2:**
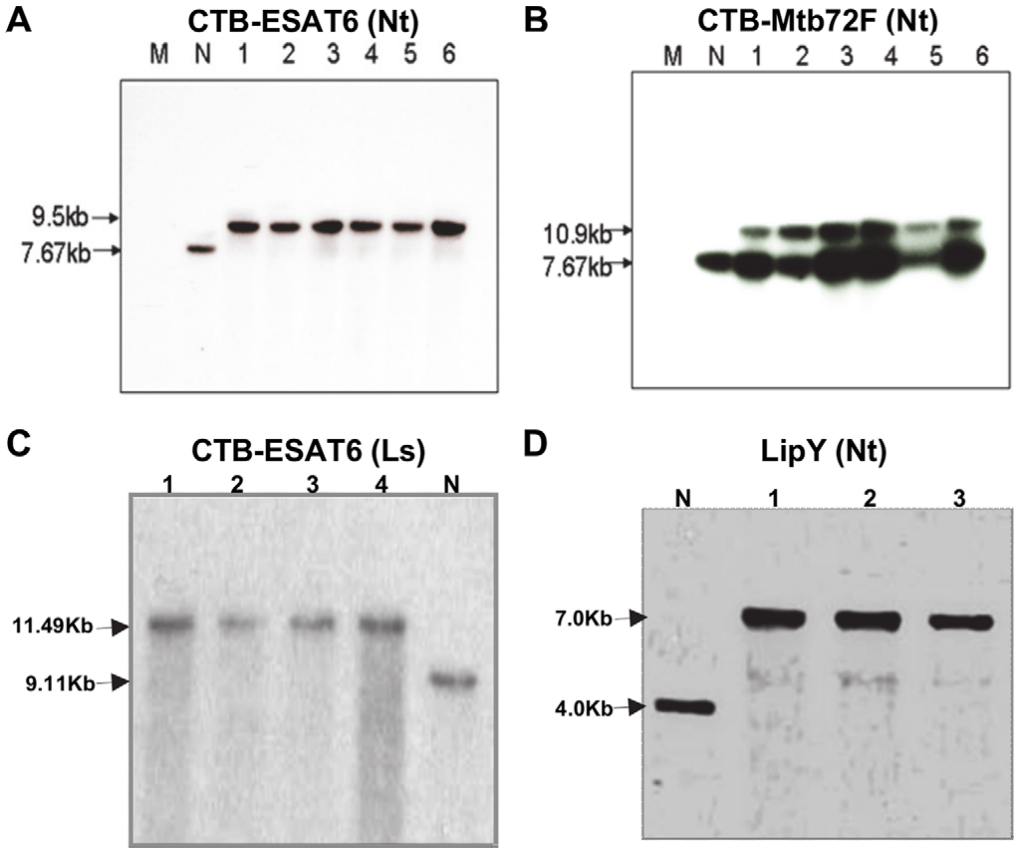
Southern blot analysis to evaluate site-specific transgene integration and homoplasmy in transplastomic plants. An autoradiograph of Southern blot hybridized with flanking sequence probe comprising of *trn*I and *trn*A gene sequences. A, Homoplasmy in CTB-ESAT6 tobacco (Nt) transplastomic lines. B, Heteroplasmy in CTB-Mtb72F tobacco (Nt) transplastomic lines. C, Homoplasmy in CTB-ESAT6 lettuce (Ls) transplastomic lines. D, Homoplasmy in LipY tobacco (Nt) transplastomic lines. Lane N, untransformed plant; Lane 1 to 6, transplastomic lines; Lane M, 1 Kb+ DNA ladder.

Following PCR analysis, transplastomic plants were subjected to two additional rounds of selection (second and third) to promote homoplasmy. Southern blot analysis was performed to determine homoplasmy or heteroplasmy and to supplement the PCR confirmation of site-specific transgene integration. The flanking sequence probe allowed detection of site-specific integration of the gene cassette into the chloroplast genome as it hybridizes with the *trn*I and *trn*A genes. The transformed chloroplast genome digested with H*ind*III produced fragments of 9.5 kb for pLD-CTB-ESAT6 (Figure 2A), 10.9 kb for pLD-CTB-Mtb72F (Figure 2B) and 11.49 kb for pLsDV-CTB-ESAT6 (Figure 2C), when hybridized with flanking sequence probe. The untransformed tobacco and lettuce chloroplast genome digested with *Hind*III produced a 7.67 kb fragment and 9.11 kb fragment respectively confirming that these plants lacked foreign genes (Figure 2A–2C). The untransformed tobacco digested with *Sma*I generated a 4.0 kb fragment whereas a ∼7.0 kb fragment was detected in LipY transformed chloroplast genomes (Figure 2D). The hybridization with flanking sequence probe revealed if homoplasmy of the chloroplast genome was achieved through several rounds of selection. Absence of 4.0 kb, 7.67 kb and 9.11 kb fragment in LipY tobacco, CTB-ESAT6 tobacco and lettuce transplastomic lines respectively confirmed homoplasmy (within the detection limits of Southern blot) and site-specific integration of foreign genes into the chloroplast genome (Figure 2A, 2C and 2D). However, for CTB-Mtb72F transplastomic plants, homoplasmy could not be achieved even after several rounds of selection on stringent selection medium containing spectinomycin, as both 10.9 kb and 7.67 kb fragments corresponding to transformed and untransformed genomes were detected in Southern blots (Figure 2B). Plants confirmed by Southern analysis were transferred to jiffy pellets and placed in 16 hr light/8 hr dark cycle incubator for acclimatization. Later, the plants were transferred to the greenhouse for maturation and seeds were collected. Tobacco transplastomic plants showed no visible difference when compared with the wild type plants and maintained normal growth and morphology in our experimental conditions (data not shown). On the other hand, lettuce T_0_ CTB-ESAT6 transplastomic plants showed detrimental signs of development with delayed growth. However, T_1_ plants were healthy and showed normal growth patterns (data not shown).

Seeds collected from CTB-ESAT6 T_0_ transplastomic and untransformed lettuce plants were germinated on ½ MS medium with spectinomycin in the same plate. All T_1_ CTB-ESAT6 lettuce seeds germinated and developed into uniformly green plants (data not shown). This lack of Mendelian segregation of genes confirmed maternal inheritance of transgenes. None of the untransformed seeds germinated on the selection media whereas all untransformed seeds germinated when placed on media without the selection agent. After prolonged periods of germination, few seeds germinated and bleaching of germinated seedlings was observed in untransformed seeds. Further, after transfer to the greenhouse, all T1 plants flowered, set seeds and showed similar phenotype when compared to untransformed plants.

### Western Blot Analysis for Detection of Foreign Protein in Transplastomic Plants

**Figure 3:**
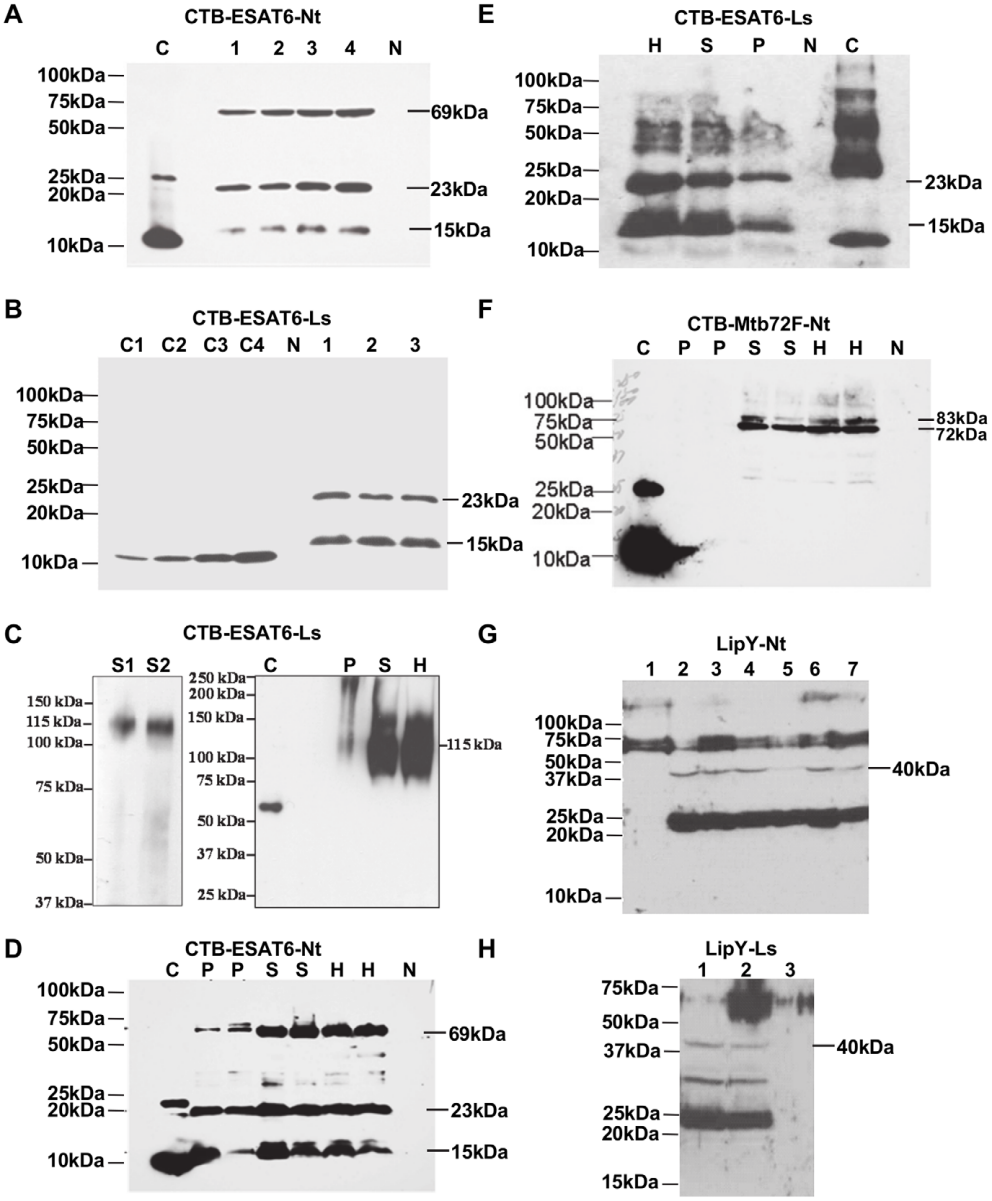
Western blot analysis to detect expression of recombinant proteins in transplastomic plants. Immunoblots with anti CTB antibody showing presence of expected size fusion protein band in CTB-ESAT6 tobacco (A) and CTB-ESAT6 lettuce (B) transplastomic lines. Immunoblots showing pentameric CTB-ESAT6 fusion protein detection with protein extracted under non-reducing/non-denaturing conditions and loaded on SDS-PAGE without boiling (C). Immunoblots showing distribution of recombinant fusion protein in the homogenate, supernatant and pellet fractions of leaf extract from CTB-ESAT6 tobacco (D), CTB-ESAT6 lettuce (E) and CTB-Mtb72F tobacco (F) transplastomic plants. (N, Untransformed; H, homogenate; S, S1 & S2: supernatant; P, pellet; 1 to 4, transplastomic plants; C, purified bacterial CTB standard; C1, C2, C3 & C4: CTB standards 25, 50, 75, 100 ng for densitometry). Immunoblot using rat anti-LipY antibody showing presence of expected band in LipY transplastomic tobacco plants (G). Lane1, untransformed; Lanes 2 to 7, transformants. Immunoblot using rat anti-LipY antibody showing presence of expected band in LipY transplastomic lettuce plants (H). Lane 1, Supernatant fraction of leaf extract from transplastomic plant; Lane 2, Homogenate fraction of leaf extract from transplastomic plant; Lane 3, untransformed.

In order to evaluate protein solubility, expression of the fusion proteins ESAT-6 and Mtb72F was analyzed by immunoblots with distinct extraction fractions (homogenate, supernatant and pellet) of leaf extracts using anti-CTB polyclonal antibody. Under reducing conditions, blots probed with anti-CTB polyclonal antibody revealed full-length fusion protein with apparent molecular mass of 23 kDa for tobacco CTB-ESAT6 (CTB-ESAT6-Nt, Figure 3A). Additional 69 kDa protein was also detected indicating the trimeric form of the fusion protein in tobacco chloroplasts (Figure 3A). In lettuce CTB-ESAT6 (CTB-ESAT6-Ls), the monomeric form of fusion protein corresponding to 23 kDa was observed under reducing conditions (Figure 3B). The trimeric 69 kDa form of CTB-ESAT6 was not detected in lettuce probably due to differences in the conditions between lettuce and tobacco leaf extracts. During the extraction of proteins from leaves, denaturing and reducing agents disrupt the trimeric and other oligomeric forms of CTB-ESAT6 fusion protein into the monomeric form. Under these conditions, fusion proteins are exposed to proteases and could result in cleavage. Exclusion of reducing and denaturing agents from plant extraction buffer showed only the pentameric structure of the CTB-ESAT6 fusion protein, without any cleaved products (Figure 3C). Under reducing conditions, other oligomeric forms of CTB-ESAT6 fusion protein were not detected due to their disruption into monomers. Under fully denatured and reduced conditions, only monomeric protein band is expected instead of disulfide bonded dimers or other oligomeric forms. Depending on the nature of CTB fusion protein, only monomeric fusion protein or dimers, trimers, tetramers and pentamers of the CTB fusion protein have been observed previously [55]–[57].

Analysis of different extraction fractions of fusion protein CTB-ESAT6 from both tobacco and lettuce indicated that most of the fusion protein existed in the soluble form. Some of the fusion protein was also detected in pellet under our experimental conditions (Figure 3D & 3E). In contrast to CTB-ESAT6, CTB-Mtb72F existed only in the soluble form and no fusion protein was detected in pellet under our experimental conditions (Figure 3F). Both tobacco and lettuce CTB-ESAT6 plants showed an additional low molecular weight band of about 15 kDa with anti-CTB antibody (Figure 3A & 3B). This fragment might have been formed by proteolytic cleavage of fusion protein. Since, the band is larger than CTB protein (11.6 kDa); cleavage should be within ESAT6. In CTB-Mtb72F (CTB-Mtb72F-Nt) tobacco plants, immunoblots with CTB antibody revealed the expected 83 kDa fusion protein besides a ∼72 kDa protein band which is due to cleavage at the C-terminal end of full length protein in the Mtb72F region (Figure 3F). Several CTB-fusion proteins have been expressed in chloroplasts and none of them showed cleavage within the CTB protein [32], [55]–[58]. The Peptide Cutter analysis tool from the ExPASy server [59] was used to predict protein cleavage sites in ESAT-6 and Mtb72F sequence. Peptide Cutter identified several cleavage sites closer to N-terminus of ESAT-6 (∼25 amino acid) indicating a higher probability of cleavage in ESAT-6 protein. Various protease cleavage sites were also detected in C-terminus of the Mtb72F sequence. However, cleavage of fusion protein occurs only during the extraction of proteins from leaves for western blot analysis under denaturing and reducing conditions. Under these conditions, fusion protein is exposed to proteases. As shown in CTB-ESAT6 (Ls) immunoblot, fusion protein remained in the pentameric form and didn’t show any cleavage when denaturing and reducing agents were excluded from plant extraction buffer (Figure 3C).

The immunoblot analysis of LipY transplastomic tobacco (LipY-Nt) and lettuce (LipY-Ls) plants with rat anti-LipY (ΔPE) antibody showed non-specific binding at ∼70 kDa in all samples including the transformants and untransformed plants (Figure 3G and 3H). Only transformed plants showed a ∼40 kDa expected size band along with ∼25 kDa band which might have formed due to the proteolytic cleavage of LipY protein in both lettuce and tobacco. Similar to CTB-ESAT6, the LipY protein existed in both soluble and insoluble fractions (Figure 3H).

### Quantification of CTB-ESAT6 and CTB-Mtb72F in Tobacco Plants Using ELISA

**Figure 4:**
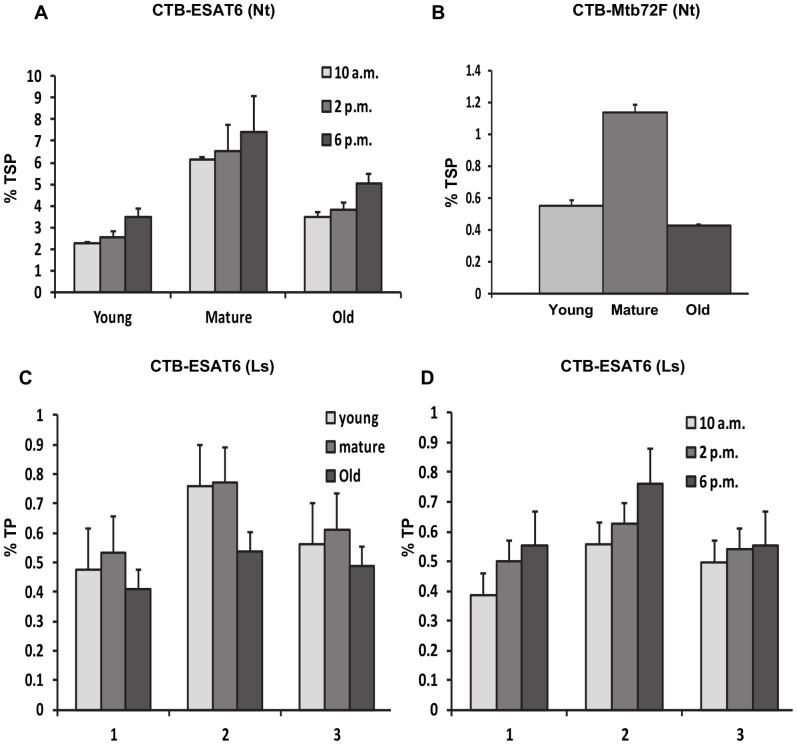
Quantitation of vaccine antigens in transplastomic lines at different developmental stages and harvesting time. A, Expression levels of tobacco CTB-ESAT6 (Nt) protein in percent total soluble protein (TSP) as a function of leaf age and harvesting time under normal growth conditions determined by ELISA. B, Expression levels of tobacco CTB-Mtb72F (Nt) protein in percent TSP in young, mature and old leaves collected from the transplastomic lines by ELISA. Expression levels of CTB-ESAT6 (Ls) protein in lettuce transplastomic lines in percent total protein (TP) by densitometry at different leaf developmental stages (C) and at various time of harvest (D). 1 to 3, transplastomic lines. Expression levels of CTB-ESAT6 (Nt) and CTB-Mtb72F (Nt) are reported as percentage of the total soluble protein whereas CTB-ESAT6 (Ls) is in percentage of the total protein. Initially, ELISA was performed to quantify CTB-ESAT6 (Nt) in the total soluble protein but subsequent analysis of pellet, supernatant and homogenate fractions of leaf extracts detected fusion protein in the insoluble fraction. Therefore, CTB-ESAT6 (Ls) fusion protein was estimated based on percentage of total leaf protein. The CTB-Mtb72F fusion protein was quantitated based on percentage of the total soluble protein as the fusion protein existed only in the soluble fraction. Data presented is mean ± SD of triplicate experiments. Protein was extracted from three independent plants and amount of CTB-fusion protein was quantified either by ELISA or by densitometry on western blots. Three independent measurements were made to quantify expression levels of fusion protein and a mean ± SD value was plotted on the graph.

Expression levels of the fusion protein CTB-ESAT6 in transplastomic tobacco plants at different developmental stages and time of leaf harvest were quantified by ELISA using leaf protein extracts prepared from young, mature and old leaves harvested at 10 AM, 2 PM and 6 PM. Under normal illumination conditions (16 h of light and 8 h of dark), the highest expression level of up to 7.5% of total soluble protein (TSP) was observed in mature leaves harvested at 6 PM, whereas in young and old leaves the expression levels reached up to 3.4% and 5% of TSP at same harvesting time (Figure 4A). The large accumulation of CTB-ESAT6 (Nt) in mature tobacco leaves probably resulted from higher number of well-developed chloroplasts and the high copy number of chloroplast genomes. Likewise, decrease in CTB-ESAT6 expression in young and old leaves could be due to less number of developed chloroplasts and degradation of proteins during senescence. Accumulation of CTB-ESAT6 (Nt) increased over time during the day and reached the highest at 6 PM. (Figure 4A). This could be attributed to 5′ Utr and the *psb*A promoter that enhances translation in light.

Although homoplasmic CTB-Mtb72F transgenic plants could not be obtained in our experimental conditions, we still quantified the expression of CTB-Mtb72F fusion protein in tobacco. The accumulation of the fusion protein reached up to 1.1% of TSP in mature leaves under the normal illumination even in highly heteroplasmic lines (Figure 4B). The observed expression levels are still much higher than other plant systems expressing TB vaccine antigens reported in published literature [34].

### Quantification of CTB-ESAT6 Fusion Protein in Transplastomic Lettuce Plants by Densitometry

Immunoblots of different extraction fractions of CTB–ESAT6 lettuce transplastomic plants indicated the presence of fusion protein in both supernatant and pellet (Figure 3E). Therefore, CTB-ESAT6-Ls expression levels were determined on western blots by comparing the homogenate fraction with known quantities of purified CTB protein and analyzing them by spot densitometry. Linearity of the standard curve was established using 25, 50 and 75 ng of purified CTB, enabling the estimation of CTB-ESAT6 expression. In lettuce, CTB-ESAT6 accumulated up to 0.75% of total leaf protein (Figure 4C). Mature leaves showed highest expression followed by young leaves (Figure 4C). Old leaves showed lowest level of expression probably due to senescence and proteolytic activity. Similarly different time points of harvest were analyzed using densitometry. An increasing trend from morning to evening with highest expression levels of fusion protein at 6 PM was observed (Figure 4D).

**Figure 5:**
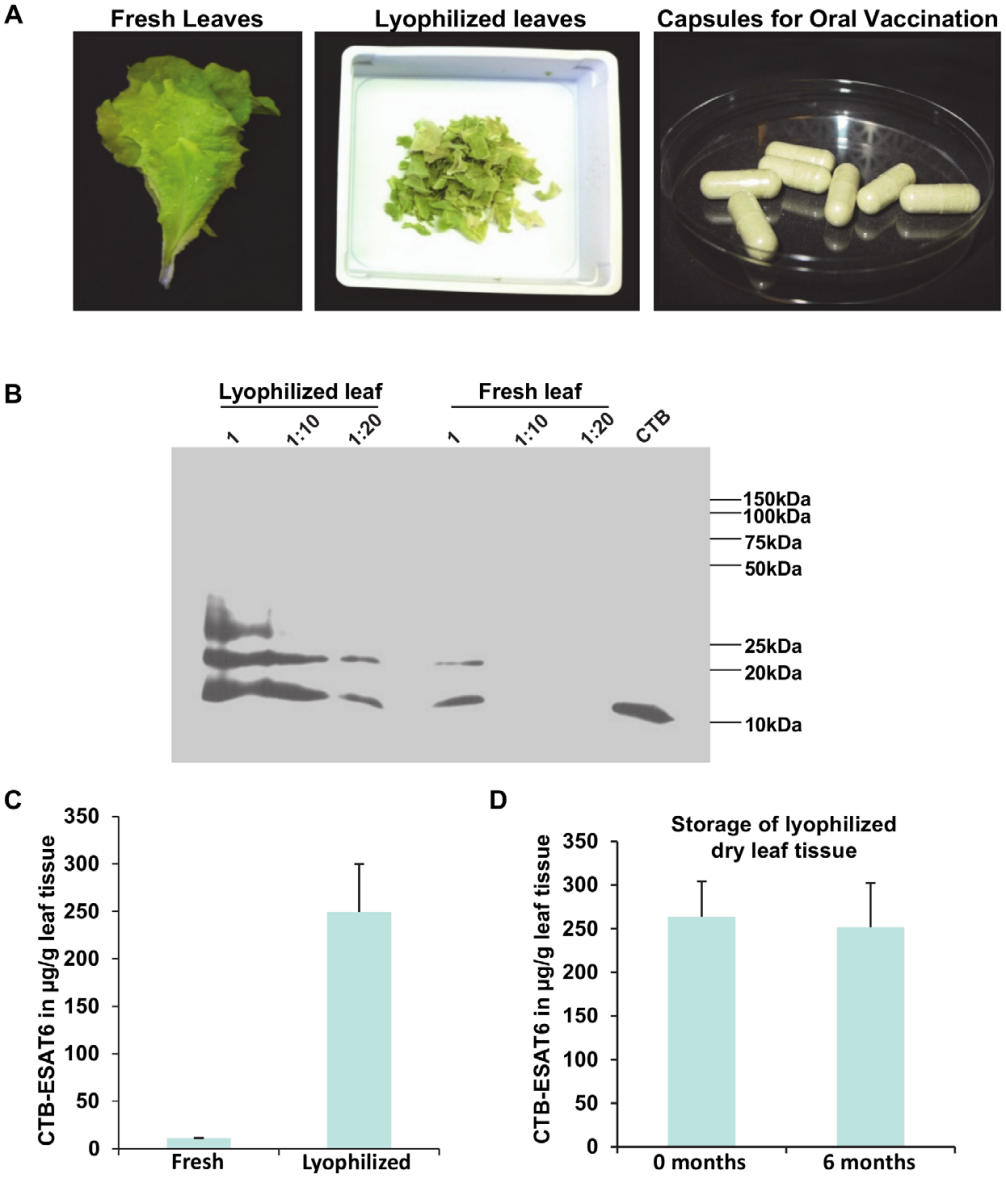
Lyophilization of CTB-ESAT6 lettuce leaves for long term storage and enrichment of antigenic fusion protein. A, Preparation of capsules containing lyophilized powdered lettuce leaves expressing CTB-ESAT6 fusion protein for use as oral vaccine. B, Immunoblot comparing amount of CTB-ESAT6 fusion protein in fresh and lyophilized lettuce leaves. Equal amount of lyophilized and fresh leaves was used for protein extraction and equal volume of protein extract was loaded with dilutions of 1x, 10x and 20x; CTB, 50 ng CTB standard. C, CTB-ESAT6 expression levels in µg/g of fresh and lyophilized leaves. D, CTB-ESAT6 protein concentration (µg/g) in lyophilized leaves stored 0–6 months at room temperature. Error bars represent standard deviation of mean.

Lyophilization of CTB-ESAT6 expressing lettuce leaves was performed to facilitate storage at room temperature. In addition, an increase in the amount of antigen per gram of leaf material was observed. After lyophilization, leaves were reduced to 5–8% of their fresh weight thereby increasing the CTB-ESAT6 antigenic content per gram of leaf material. Lyophilized material was powdered and packaged into capsules (size zero, Torpaq Inc.) to be delivered as oral vaccine (Figure 5A). Analysis of lyophilized material on immunoblots with anti-CTB antibody revealed same polypeptides as obtained with fresh leaf material (Figure 5B). The undiluted and 10-fold diluted lyophilized leaf samples didn’t show any difference in 23 kDa band or cleaved 15 kDa polypeptide intensities in accordance with the dilution factor, indicating saturation of signal in the undiluted sample. The 20 fold diluted lyophilized sample showed that there was a 22 fold increase in antigen content per gram of lyophilized leaves, when compared to fresh leaves (Figure 5B and 5C). There was no significant difference in CTB-ESAT6 amount in lyophilized leaves stored for six months when compared with latest lyophilized leaves suggesting that lyophilized material was stable even after 6 months of storage at room temperature (Figure 5D).

### Binding of Chloroplast-derived CTB-ESAT6 to the GM1-ganglioside Receptor

**Figure 6:**
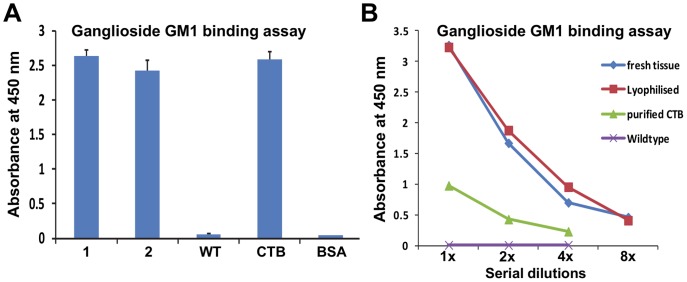
Functional evaluation of CTB-ESAT6 fusion protein in fresh and lyophilized lettuce leaves. A, Ganglioside GM1 ELISA binding assay of CTB-ESAT6 derived from total leaf protein (TLP) of transplastomic plants. 1, 2∶15 µg TLP from CTB-ESAT6 transplastomic plants; WT, 15 µg TLP from untransformed lettuce plant; CTB, purified CTB standard; BSA, 50 ng bovine serum albumin. B, Ganglioside GM1 ELISA binding assay of CTB-ESAT6 from fresh and lyophilized lettuce leaves. Fresh and lyophilized CTB-ESAT6 lettuce leaf extracts starting from 15 µg equal concentrations and with decreasing two fold dilutions were analyzed for binding assay; Wild type, untransformed leaf extract; Purified CTB, 10 ng purified CTB standard.

To evaluate whether CTB-ESAT6 fusion protein produced in lettuce chloroplasts retained its biological function of binding to the GM1 receptor, GM1- binding ELISA assay was performed. A pentameric structure of CTB protein is required for binding to its receptor GM1- ganglioside in vivo [60], [61]. CTB- ESAT6 plant extract along with purified CTB protein showed strong binding affinity to GM1 (Figure 6A & 6B). Untransformed plants and bovine serum albumin (BSA) didn’t show binding to the GM1 receptor (Figure 6A & 6B). Effect of lyophilization on the binding ability of CTB-ESAT6 fusion protein was also tested with GM1 ELISA binding assay. Extracts from CTB-ESAT6 lyophilized lettuce leaves effectively bound to the GM1-ganglioside receptors (Figure 6B). Further, serial dilution of extract from fresh, lyophilized leaves and purified CTB showed decrease in absorbance accordingly (Figure 6B). GM1 binding of CTB-ESAT6 fusion protein from lyophilized and fresh lettuce leaves indicates that the CTB in the fusion protein has its native pentameric structure and has not been disrupted by its fusion to ESAT6 or by the lyophilization process. Binding to GM1 receptor is essential for antigen uptake in the gut.

### Evaluation of Pore Formation in Red Blood Cell Membranes Using Hemolysis Assay

**Figure 7:**
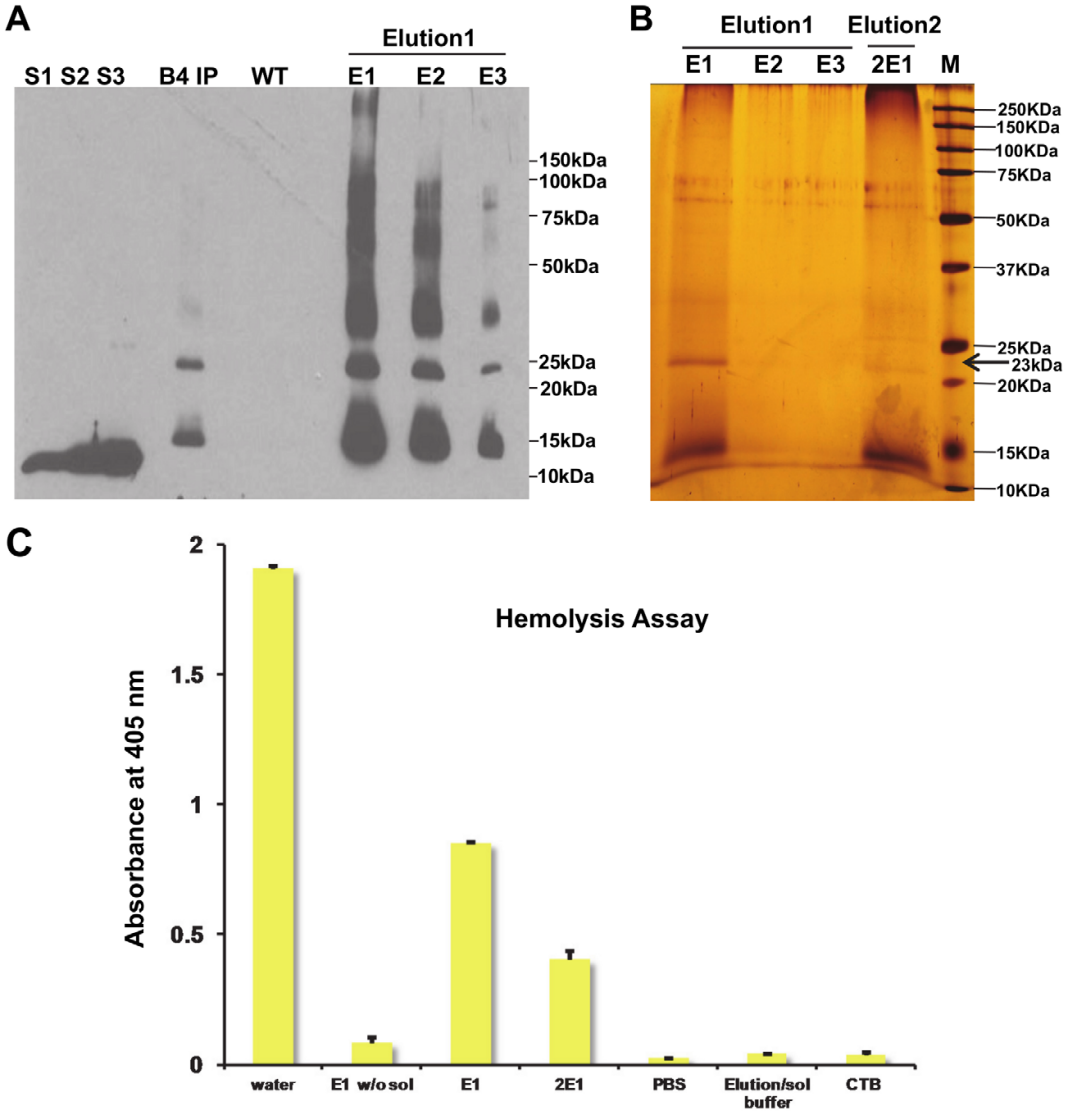
Hemolysis assay with antibody affinity purified CTB-ESAT6. A, Immunoblot with CTB antibody to confirm the presence of CTB-ESAT6 fusion protein. S1, S2, S3∶12.5, 25, 37.5 ng CTB standard; E1, E2, E3∶5 µl of different elution fractions; B4IP, 10 µg of total protein extract from CTB-ESAT6 transplastomic line before purification; WT, 10 µg of total protein extract from untransformed lettuce plant. B, Silver stained gel of affinity purified CTB-ESAT6 fusion protein. Elution1, 1st elution; Elution2, 2nd elution; E1, E2, E3, 2E1∶20 µl of different elution fractions of CTB-ESAT6; M, 0.4 µl protein standard marker. C, Hemolysis assay of ESAT6 with purified fusion protein CTB-ESAT6. Water causes complete hemolysis; E1 w/o sol, 40 µg/ml partially purified CTB-ESAT6 protein after 1st elution without solubilization; E1, 40 µg/ml partially purified CTB-ESAT6 protein after 1st elution with solubilization; 2E1, 20 µg/ml partially purified CTB-ESAT6 protein after 2nd elution with solubilization; PBS, phosphate buffer saline control; Elution/sol buffer, elution buffer used for protein elution/solubilisation; CTB, 40 µg/ml purified CTB. Standard deviations are represented by error bars of mean. Values are of 2 independent experiments in duplicates.

Lettuce expressing CTB-ESAT6 protein is an oral edible vaccine for TB; therefore there is no need to perform purification of antigens. Hence, a tag such as histidine was not incorporated in the coding sequence. To perform hemolysis assay, purification of CTB-ESAT6 fusion protein is necessary. Therefore, CTB–ESAT6 fusion protein was purified from lettuce plants using immunoaffinity purification with CTB antibody. Western blot analysis of purified protein detected multiple bands corresponding to monomeric 23 kDa, cleaved 15 kDa and aggregates or multimers of >23 kDa molecular weight (Figure 7A). Based on densitometry analysis, up to 40 µg/ml of 80% pure CTB–ESAT6 was obtained and this concentration was further confirmed by ELISA. Silver staining of purified protein showed two polypeptides of ∼23 kDa and ∼15 kDa (Figure 7B). The trimeric 69 kDa fragment was not observed in silver stained gels, probably due to lower concentration when compared with polypeptides of ∼23 kDa and ∼15 kDa. In addition, denaturing and reducing conditions should disrupt the trimeric form of CTB-ESAT6 fusion protein into the monomeric form. Presence of ∼15 kDa protein band showed that the cleavage occurred in the ESAT6 protein. The purified CTB-ESAT6 protein was used for hemolysis assay.

*Mycobacterium tuberculosis* uses ESX-1 secretion system to export virulence proteins during infection. ESAT-6 is one of the secreted proteins in ESX-1 system and has been reported to play a role in the escape of *Mycobacterium* from the phagolysosome [62] by membrane pore formation [51]. Purified ESAT-6 has been shown to cause dose dependent hemolysis in red blood cells by membrane pore formation [51]. The red blood cell lysis by pore forming proteins occurs by osmotic shock. The hemolytic effect of plant-derived partially purified ESAT6 was investigated on red blood cell membranes after two hour incubation. Hemolysis was measured by the absorbance (O.D.) of the red blood cell supernatant which contains hemoglobin. Partially purified CTB-ESAT6 when solubilized resulted in dose dependent hemolysis. CTB-ESAT6 formed aggregates in its native form and hence was solubilized to dissociate the oligomeric protein. Purified protein without solubilisation did not cause hemolysis at a concentration of 40 µg/ml (Figure 7C). After solubilisation, CTB-ESAT6 at 40 µg/ml protein concentration caused partial hemolysis of red blood cells with an absorbance of 0.85. Decrease in absorbance to 0.4 was observed when the protein was diluted two fold (Figure 7C). This indicated that the fusion did not modify ESAT-6 protein’s ability to form pores and lysed red blood cell membranes in a dose-dependent manner. Red blood cells incubated with water resulted in complete lysis (absorbance of 1.9) as the cells swell and burst due to movement of water into cells (hypotonic solution). Red blood cells incubated in PBS solution were intact and therefore no absorbance was detected (Figure 7C). Also, purified CTB protein suspended in PBS was analyzed to see if there was any hemolytic effect shown by CTB (concentration of 40 µg/ml) but no effect was observed.

## Discussion

Recent advances in genetic engineering have increased the potential of using plant chloroplasts for high level of foreign protein expression, cost-effectiveness, scalability and safety of recombinant biopharmaceuticals and vaccines [37], [63], [64]. To our knowledge, this is the first report of expression of TB vaccine antigens in chloroplasts. In this study, we expressed ESAT6, Mtb72F and LipY, three ideal candidate antigens for TB vaccine development in tobacco and lettuce chloroplasts. Results show an efficient and stable expression of the recombinant fusion protein CTB-ESAT6, CTB-Mtb72F and LipY protein in chloroplasts. The main factors that are essential for practical development of oral plant vaccines are sufficient expression level of antigens in the system to meet adequate dosage (leaf material) required for immunization, stability and storage in addition to vaccine efficacy. Chloroplast expression system is well suited for production of TB vaccine antigens to achieve these goals.

In relation to antigen expression, CTB-ESAT6 tobacco plants reached up to 7.5% of the TSP in the mature transgenic tobacco leaves under normal illumination. The recombinant CTB-Mtb72F accumulated up to 1.1% of TSP, which could be due to heteroplasmy observed in transplastomic plants. The CTB-Mtb72F plants were heteroplasmic even after additional rounds of selection probably due to the instability caused by improper folding of the fusion protein as GPGP hinge by itself was not capable of preventing steric hindrance, as reported previously for CTB-FIX [56]. In the greenhouse, CTB-Mtb72F heteroplasmic plants were grown without antibiotic selection. Leaves harvested from the greenhouse grown plants showed expression and accumulation of CTB-Mtb72F (Figure 3F and 4B) confirming transgene expression in the absence of any selection. Persistence of such heteroplasmy in subsequent generations of transplastomic lines has been observed previously [65]. CTB-Mtb72F and LipY were expressed in LAMD, a low nicotine variety of tobacco that may be a suitable system for oral delivery of vaccine antigens. The CTB–ESAT6 lettuce transplastomic plants have modest expression levels of 0.75% of total leaf protein (TP). In comparison to the tobacco system, earlier studies have shown lettuce to have lower expression levels with some antigens. In case of CTB–AMA1 fusion protein, a higher level of expression of ∼13% TSP was found in tobacco when compared to ∼7% TSP in lettuce [32]. The variation in expression levels of recombinant proteins can be due to many factors including nature of protein, plant system used, environmental conditions, protein stability in chloroplasts and regulatory elements present in expression cassettes [64]. Since tobacco system expressed higher levels of CTB-ESAT6, this variation could be due to protein instability in lettuce chloroplasts.

Our results indicated that approximately 950 µg of CTB-ESAT6 protein can be obtained per gram fresh weight of mature tobacco leaves under normal illumination. Accordingly, 80mg of CTB-ESAT6 can be obtained from a single tobacco plant and a total of 1.92 kg can be produced from an acre of land based on three cuttings in a year. For some commercial tobacco cultivars whose yield are almost 20 fold more than that of experimental cultivar Petite Havana [66], it is expected to produce more vaccine antigens at lower cost of vaccination for larger populations. The CTB-ESAT6 lettuce plants expressed 11.2 µg/g of antigen (fresh weight mature leaf) whereas lyophilization increased the yield 22 fold as the antigenic content built up to 249 µg/g of lyophilized leaves. There is no need to produce 22 times more fresh leaf for each dose but lyophilization reduces the biomass requirement for packaging by 22-fold. Therefore, adequate amounts of transgenic protein are available for oral delivery. In human trials for TB subunit vaccine ESAT-6, 50 µg of vaccine antigen was injected intramuscularly [21]. So if we hypothesize that 50 µg were to be orally delivered, based on quantification of antigenic content, only 200 mg of lyophilized material would be needed. Lyophilized lettuce expressing antigens has been successfully used in orally delivered plant vaccine animal studies [67], [68]. Accordingly, only 9 mg of lyophilized tissue expressing hepatitis B surface in the form of tablets was orally fed to mice with an interval of 60 days between priming and boosting to induce an immune response. Tablets or capsules could provide the means to antigen standardization and dosage control and are easy for distribution. Expression level of any vaccine antigen, including Mtb72F will vary depending upon the developmental stage, duration of illumination, time of harvest, etc. Therefore, each batch of leaves must be quantified after harvest and lyophilization and then packaged into capsules so that desired dosage is maintained and delivered. Long term stability of antigens after storage at room temperature should facilitate in maintaining proper dosage of antigens in capsules. Therefore, dosage is not determined by expression level in leaves but it should be controlled in capsules in each batch of production.

Lettuce (*Lactuca sativa)* was chosen as an alternative to tobacco for expression of TB vaccine antigens because it is a leafy vegetable and has great commercial value. The lettuce CTB-ESAT6 plants showed modest expression levels, which can be overcome with lyophilization, a process of freeze drying. Since lettuce has high water content (95%), it can be freeze dried to a greater magnitude than other plant leaves. Lettuce is a relatively easy plant to grow with no special conditions except cooler environment. Since it is a leafy vegetable, more antigen could be concentrated by lyophilization of leaf tissue. Furthermore, lyophilization process has been shown to remove microbes as opposed to the fresh lettuce leaves which harbored ∼6000 cfu/g microbes [57], [69]. The CTB-ESAT6 protein was stable in lyophilized material stored at room temperature for six months. (Figure 5D). This stability of antigens in plant tissue could help in eliminating cold chain during storage and distribution required for conventional vaccines. Also, harvesting leaves before appearance of flowers maximizes transgene containment.

The ability of CTB-ESAT6 to form pentamers facilitates binding to GM1 ganglioside receptors and increased antigen uptake. Activity of ESAT-6 has been characterized as a cytolysin that can disrupt lipid bilayers [70] and pore formation in cell membranes [51]. Hemolysis assay established the ability of partially purified CTB-ESAT6 to create partial lysis of red blood cells. Plant derived CTB-ESAT6 has been shown to retain its biological activity and has potential to be an effective oral vaccine.

Production of an oral subunit vaccine against tuberculosis in chloroplast is a promising strategy to overcome the cost constraints such as production, purification, processing, cold storage, transportation and delivery linked to large vaccination campaigns, especially with the increasing TB incidence in some developing countries where there is a dire need of such vaccines. The estimated costs associated with production and delivery of the recombinant proteins in bacterial, insects or mammalian cells is much higher when compared to plants [71]. Easy and unlimited scalability of protein production and absence of the viral contamination can make plant-derived biologicals, economical and safer for large-scale production [37], [72], [73]. Production cost of antigen in GMP facility greenhouse has been estimated to be 100–220 fold less than other current production systems [74]. However, oral delivery of antigens bioencapsulated in plant cells eliminates the highly expensive purification, cold storage, transportation and sterile injections, significantly reducing their cost.

BCG, the only current TB vaccine is delivered parenterally and shows variable protection. There is a need for alternative routes of vaccine delivery targeting the mucosa which is the site of invasion of *Mycobacterium tuberculosis*. Oral vaccines confer both mucosal (IgA) and systemic immune response [75]. In order to combat the TB disease with a safer vaccine, subunit vaccines are being pursued with recombinant TB antigens. Many TB vaccine candidates are in clinical trials such as recombinant BCG candidates VPM1002, rBCG30 and there are booster vaccine candidates such as MVA85A, Crucell Ad35, which employ non virulent viral vectors for delivery [76]. A key latent phase antigen, α-crystallin, has been analyzed in guinea pigs to boost the BCG induced immunity by BCG prime-DNA boost treatment and conferred strong protection when compared with BCG vaccination [11]. Currently, an ideal strategy to combat TB is to administer BCG to infants and later on boost immunity by effective subunit booster vaccine. The production of CTB-ESAT6, CTB-Mtb72F and LipY in plant chloroplasts provides suitable plant based oral booster vaccines. Following priming with BCG, oral boosters of plant based TB vaccine antigens has potential to effectively provoke mucosal as well as systemic immunity. Chloroplast-derived vaccine candidates including CTB-AMA1, CTB-MSP1 and F1-V fusion protein has been effectively employed in a subcutaneous prime and oral boost treatment regimen [29], [32]. In addition, after challenge with fatal dosage of aerosolized Y. pestis, orally immunized mice showed enhanced immunity and protection when compared with subcutaneous injections [29]. Moreover, vaccine antigens produced in plants are bioencapsulated and protects the antigen from hostile acidic environment of stomach upon oral delivery. After reaching the gut, microbes colonizing gut digest plant cell walls and vaccine antigens are presented to the immune system of the gut.

For evaluation of the TB vaccine antigens expressed in chloroplasts, studies will be performed in future to test their immunogenicity in suitable animal models. Lettuce, an edible crop plant and LAMD, low nicotine tobacco variety are ideal systems for oral delivery of CTB-ESAT6, LipY and CTB-Mtb72F and can pave the way for future cost-effective oral TB booster vaccines.
